# Long-term feeding of high-fat plus high-fructose diet induces isolated impaired glucose tolerance and skeletal muscle insulin resistance in miniature pigs

**DOI:** 10.1186/s13098-017-0281-6

**Published:** 2017-10-13

**Authors:** Meng-Chieh Hsu, Mu-En Wang, Yi-Fan Jiang, Hung-Chang Liu, Yi-Chen Chen, Chih-Hsien Chiu

**Affiliations:** 1 Laboratory of Animal Physiology, Department of Animal Science and Technology, National Taiwan University, No. 50, Ln. 155, Sec. 3, Keelung Rd., Da’an Dist., Taipei, 106 Taiwan, Republic of China; 2 Graduate Institute of Molecular and Comparative Pathobiology, School of Veterinary Medicine, National Taiwan University, No. 1, Sec. 4, Rooservelt Rd., Da’an Dist., Taipei, 106 Taiwan, Republic of China; 3 Department of Thoracic Surgery, Mackay Memorial Hospital, No. 92, Sec. 2, Chung-Shan North Rd., Zhongshan Dist., Taipei, 104 Taiwan, Republic of China

**Keywords:** Insulin resistance, Miniature pig, High fat high fructose diet, IVGTT, Impaired glucose tolerance

## Abstract

**Background:**

During the prediabetic development, the changes in β-cell function and tissue-specific insulin resistance have been described. However, there are conflicting views in insulin secretory capacity between early clinical observation and recent proposed mathematical model. On the basis of digestive and metabolic similarities with humans, swine have great potential as an animal model to investigate the progressive mechanisms of prediabetes. The aim of this study was to investigate the insulin secretory response and tissue-specific insulin resistance in a dietary-induced prediabetic porcine model.

**Methods:**

Adult male Taiwan Lee-Sung miniature pigs were randomized into two groups: (1) low-fat diet and (2) high-fat plus high-fructose diet (HFHF; 20.9% crude fat and 17.8% fructose). During the 12-month dietary intervention, body weights and blood glucose levels were measured monthly. Intravenous glucose tolerance test was used for measuring glucose tolerance and insulin secretory capacity. At the end of the experiment, liver and soleus muscle specimens were collected for ex vivo insulin sensitivity testing.

**Results:**

The results showed that the HFHF group had obesity, hyperinsulinemia, and dyslipidemia, but normal fasting glucose levels. The HFHF pigs exhibited enhanced first- and second-phase insulin secretion and high 2-h postload glucose levels in intravenous glucose tolerance test. Furthermore, the skeletal muscle specimens from the HFHF group were desensitized to insulin stimulation as shown by the lack of AKT Ser473 phosphorylation; however, the liver specimens remained a normal response.

**Conclusions:**

In conclusion, the HFHF diet-fed pigs developed isolated impaired glucose tolerance corresponding to prediabetes with an intense insulin secretory response and skeletal muscle insulin resistance.

**Electronic supplementary material:**

The online version of this article (doi:10.1186/s13098-017-0281-6) contains supplementary material, which is available to authorized users.

## Background

Prediabetes is described as a state in which an individual has a high risk for future development of diabetes. The diagnosis of prediabetes is based on a fasting plasma glucose level (100–125 mg/dl) and 2-h plasma glucose level (140–199 mg/dl) after oral glucose tolerance test. Depending on the test results, prediabetes is classified into isolated impaired fasting glucose (IFG), isolated impaired glucose tolerance (IGT), or combined IFG/IGT [1, 2]. Within numerous clinical studies in the pathology of prediabetes, the changes of insulin secretion capacity and tissue insulin sensitivity have been delineated.

Early clinical reports indicated that first-phase insulin secretory response was decreased in individuals with isolated IFG and IGT compared to individuals with normal glucose tolerance (NGT) [3–6]. Moreover, individuals with isolated IGT also showed a severely diminished second-phase insulin secretory response [5, 6]. However, a recent study proposed a mathematical model of pancreatic insulin secretion that is in disagreement with the above reports [7]. In insulin resistance associated with prediabetes, the insulin secretory response increases as a compensatory mechanism. In this model, isolated IFG had a higher first-phase insulin secretion level; isolated IGT had a higher second-phase insulin secretion level; combined IFG/IGT had higher first- and second-phase insulin secretion levels compared to NGT.

Since liver and skeletal muscle are the major tissues responsible for glucose homeostasis, the insulin resistance in different tissues and their interaction complicate the pathology of developing prediabetes. The hypothesis, skeletal muscle insulin resistance promoting the developments of non-alcoholic fatty liver disease and hepatic insulin resistance, has been proposed [8, 9]. Intramyocellular lipid accumulation causes skeletal muscle insulin resistance and further impaired glucose transport and glycogen synthesis. Under the condition of the muscle insulin resistance, the liver extends the metabolized capacity of ingested carbohydrates and promotes hepatic de novo lipogenesis [8]. In this view, the insulin resistance in skeletal muscle might occur earlier than in liver. To address this hypothesis, the tissue-specific insulin resistance was examined in the current study.

Over the last 30 years, swine have been considered a suitable animal model for diabetic and metabolic research because their pancreas morphology, gastrointestinal physiology, nutrition requirements, plasma lipid profiles and metabolism are similar to humans’. Miniature pigs have a smaller body size and are more easily handled than domestic pigs, and therefore these pigs are commonly used in biomedical studies. Several miniature pig strains, including Yucatan, Göttingen, Guizhou, and Ossabaw, have been used for the development of diabetes models [10]. It has been reported that pigs fed diets with excessive fat and energy displayed dyslipidemia, hyperinsulinemia, and obesity; however, most of these models did not exhibit severe hyperglycemia [11]. Thus, the porcine model of type 2 diabetes by dietary manipulation should be explored and examined in other strains.

The Lee-Sung pig is a strain cross-bred between Lanyu small-ear miniature pigs and Landrace pigs [12]. This strain grows to 62 kg in average weight at 21 months of age, which is similar to adult human body weight [13]. Hence, we examined could the possibility to establish a diet-induced prediabetic porcine model utilizing adult Lee-Sung pigs via long-term feeding with a high-fat plus high-fructose (HFHF) diet. Furthermore, we evaluated the changes in glucose tolerance, insulin secretory response, and tissue-specific insulin resistance in these pigs after the dietary intervention.

## Methods

### Animals and handling

Seven adult castrated Lee-Sung pigs (2-years old) were used in this study. All of animal experimental procedures were conducted in accordance with the Guide for the Care and Use of Laboratory Animals and were approved by the Institutional Animal Care and Use Committee of National Taiwan University. The animals were housed in the Experimental Farm, National Taiwan University and kept in natural dark–light cycles and ambient temperature. Pigs were allowed an acclimatization period of 2 weeks and were gradually introduced to their experimental diets. Pigs were randomly divided into two groups and fed with different diets: low-fat diet (n = 3), and high-fat plus high-fructose diet (HFHF, n = 4) containing 17.8% fructose and 21.2% crude fat (Additional file 1: Table S1), modified from Lee’s dietary formula [14]. The HFHF diet provided excess fat, sugars, and calories, but no cholesterol supplementation. Animals were fed twice daily at 7 a.m. and 5 p.m. with a restricted dietary amount (~ 2700 kcal/animal/day) in the low-fat group or ad libitum feeding (~ 5200 kcal/animal/day) in HFHF group. Water was freely accessible.

Animal weights, overnight fasting serum glucose and insulin levels were measured monthly. At the end of the experimental period, animals were sacrificed 6 h after the last meal by using the head-only electrical stunning and followed by neck exsanguination. The blood and tissue samples were collected for further analyses.

### Serum biochemistry

Blood samples were collected from the jugular vein and incubated for 1 h at room temperature to allow clotting. Serum was collected by centrifugation at 5000×*g* for 10 min and stored at − 80 °C until analyzed. Serum glucose, triglyceride, total cholesterol, alanine aminotransferase (ALT), aspartate aminotransferase (AST), and uric acid levels were assayed using a SPOTCHEM SP-4410 automatic dry chemistry analyzer (ARKRAY, Inc., Kyoto, Japan). Serum insulin levels were measured using porcine insulin ELISA kit (Mercodia AB, Uppsala, Sweden). Serum high density lipoprotein cholesterol (HDL-c) and low and very low density lipoprotein cholesterol (LDL/VLDL-c) were measured using the HDL and LDL/VLDL quantitation kit (Sigma-Aldrich, St. Louis, MO, USA). Serum non-esterified fatty acid (NEFA) was measured using the free fatty acid quantitation kit (Sigma-Aldrich).

### Quantification of insulin resistance

Insulin resistance was determined from the values of fasting blood glucose and insulin using a homeostatic model assessment (HOMA-IR) [15]. The formula is (G_b_ × I_b_)/405, where G_b_ and I_b_ are the basal (fasting) levels of glucose (mg/dl) and insulin (μU/ml) concentration. For the requirement of calculating the formula, the unit conversion factor of porcine insulin used was 1 μU/ml = 6 pmol/l.

### Intravenous glucose tolerance test

At the 9- and 12-month time points, each pig was subjected to intravenous glucose tolerance tests (IVGTT) to assess serial changes in systemic insulin sensitivity. After a 16-h fast, the pigs were sedated with Stresnil (4 mg/kg, i.m.; Janssen Pharmaceutica N.V., Beerse, Belgium) containing atropine (0.04 mg/kg, i.m.), and then anesthetized with Zoletil 50 (1.7 mg/kg, i.m.; Virbac, Carros, France). A 24G indwelling needle was inserted into the ear vein to withdraw blood samples. The pigs were injected with 50% glucose (0.5 g/kg) via the jugular vein, using a 13G 4-inch steel needle and ultrasonic equipment M7Vet (Mindray Inc., DC, USA). Blood samples were collected 5, 10, 15, 20, 25, 30, 40, 50, 60, 75, 90, 105, 120, 150, and 180 min from the end of the glucose injection for the measurement of glucose and insulin concentrations. Blood glucose concentrations were measured by glucometer ELITE XL (Bayer Co., Mishawaka, IN, USA) immediately. Serum samples were stored at − 80 °C until the measurement of insulin concentrations using a porcine insulin ELISA kit (Mercodia). Some higher insulin concentration samples were diluted three times with kit diluent and underwent further analysis. The acute insulin response (AIR) was calculated as the difference in mean insulin concentration at 5 and 10 min minus the insulin concentration at 0 min of the IVGTT. The rate of increase in insulin levels during the second phase was calculated as the mean of $\left( {{\text{Ins}}_{T} - {\text{Ins}}_{{T^{\prime\prime}}} } \right)/\left( {T - T^{\prime\prime}} \right)$, where Ins is insulin concentration, *T* is each time point from 10 to 40 min, and *T″* is the previous time point of *T*.

### Ex vivo insulin stimulation of muscle and liver tissues

The method of ex vivo tissue culture mainly referred to the previous study [16]. The fresh soleus muscle and liver tissue samples were obtained by 16G SuperCore™ semi-automatic biopsy needle (Argon medical device Inc., Plano, TX, USA) to make equivalent column size specimens. The size of each specimen was 1.2 (diameter) × 20 (length) mm; the weight was approximately 25 mg. For treating three different concentrations of insulin, three specimens were obtained from each tissue. During sampling, the specimens were immersed in cold Krebs–Henseleit bicarbonate buffer (KHB; pH 7.35, 10 mM HEPES, 24 mM NaHCO_3_, 114 mM NaCl, 5 mM KCl, 1 mM MgCl_2_, and 2.2 mM CaCl_2_) or DMEM medium (Sigma-Aldrich) without glucose for muscle or liver specimens, respectively. The muscle specimens were then transferred into fresh pre-warmed KHB containing 3 mM glucose, 7 mM mannitol, and 0.1% (w/v) bovine serum albumin (BSA) in the absence (0 nM) and presence (10 or 100 nM) of porcine insulin (Sigma-Aldrich) and incubated at 37 °C for 30 min. After incubation, the specimens were washed with PBS twice and stored at − 80 °C until analysis. For liver specimens, the buffer was substituted with DMEM medium for KHB and the procedure was the same as described above.

### Western blot analysis

For preparation of total cellular protein, 10 mg tissue samples were homogenized in cold lysis buffer containing protease inhibitor cocktail (Roche, Basel, Switzerland) and phosphatase inhibitor cocktail (Roche). The total homogenate was centrifuged at 13,000×*g* for 10 min to pellet cell debris. Protein concentration in the supernatant was determined by BCA assay (Thermo Fisher Scientific, Waltham, MA, USA). One-quarter volume of 4 × Laemmli buffer was added into the lysate that was then boiled for 5 min. The tissue extracts were then subjected to 10% sodium dodecyl sulfate polyacrylamide gel electrophoresis (SDS-PAGE), and the separated proteins were transferred to a polyvinylidene fluoride membrane. The membrane was blocked with 5% non-fat milk for 1 h and then incubated overnight at 4 °C with anti-phospho-AKT (#4060, Cell Signaling Technology, Inc., Danvers, MA, USA) antibody in TBS containing 0.1% Tween-20 (TBST) and 1% BSA. After being washed in TBST, the membrane was incubated for 1 h at room temperature with horseradish peroxidase-conjugated secondary antibody (Santa Cruz, Dallas, TX, USA) in TBST with 5% non-fat milk. After washing in buffer, signals were detected by ECL (GE Healthcare, Little Chalfont, UK) using a CCD camera system (Topbio Co. Taipei, Taiwan). For detection of total AKT as an internal control, the same membrane was incubated with stripping buffer (Thermo Fisher Scientific) for 15 min at 37 °C. After washing with TBST, the membrane was re-probed with anti-pan-AKT antibody (#4691, Cell Signaling Technology) as above. The protein expression level was determined by obtaining the signal density in a defined area using ImageJ version 1.49b (NIH, Bethesda, MD, USA). To evaluate the insulin sensitivity in each pig, the result represents the value of p-AKT^S473^/pan-AKT normalized to insulin 0 nM treatment in each pig.

### Statistical analysis

The measurements of body weight, glucose level, insulin level, HOMA-IR, and serum biochemistry at the same time point were analyzed by Student’s *t* test. Significant differences between groups were determined at the 0.05 probability level (*p* < 0.05). In the IVGTT experiments, the data of two groups at the same month were analyzed by Student’s t test. To compare the data between different month time points in the same group, paired Student’s t test was used. In the ex vivo experiments, insulin treatments in the same dietary group were subjected to one-way analysis of variance (ANOVA). When the significance was detected among dosage treatments at *p* < 0.05, the difference between treatments was evaluated by the least significant difference (LSD) test. To compare the insulin responses at the same concentration between two dietary groups, Student’s t test was used. All results are expressed as means ± SEM. All statistical analyses of data were performed using IBM SPSS Statistics version 20 (IBM Corp. Armonk, NY, USA) and Microsoft Excel (2010).

## Results

### Monthly body weights, fasting blood glucose and insulin levels

**Fig. 1:**
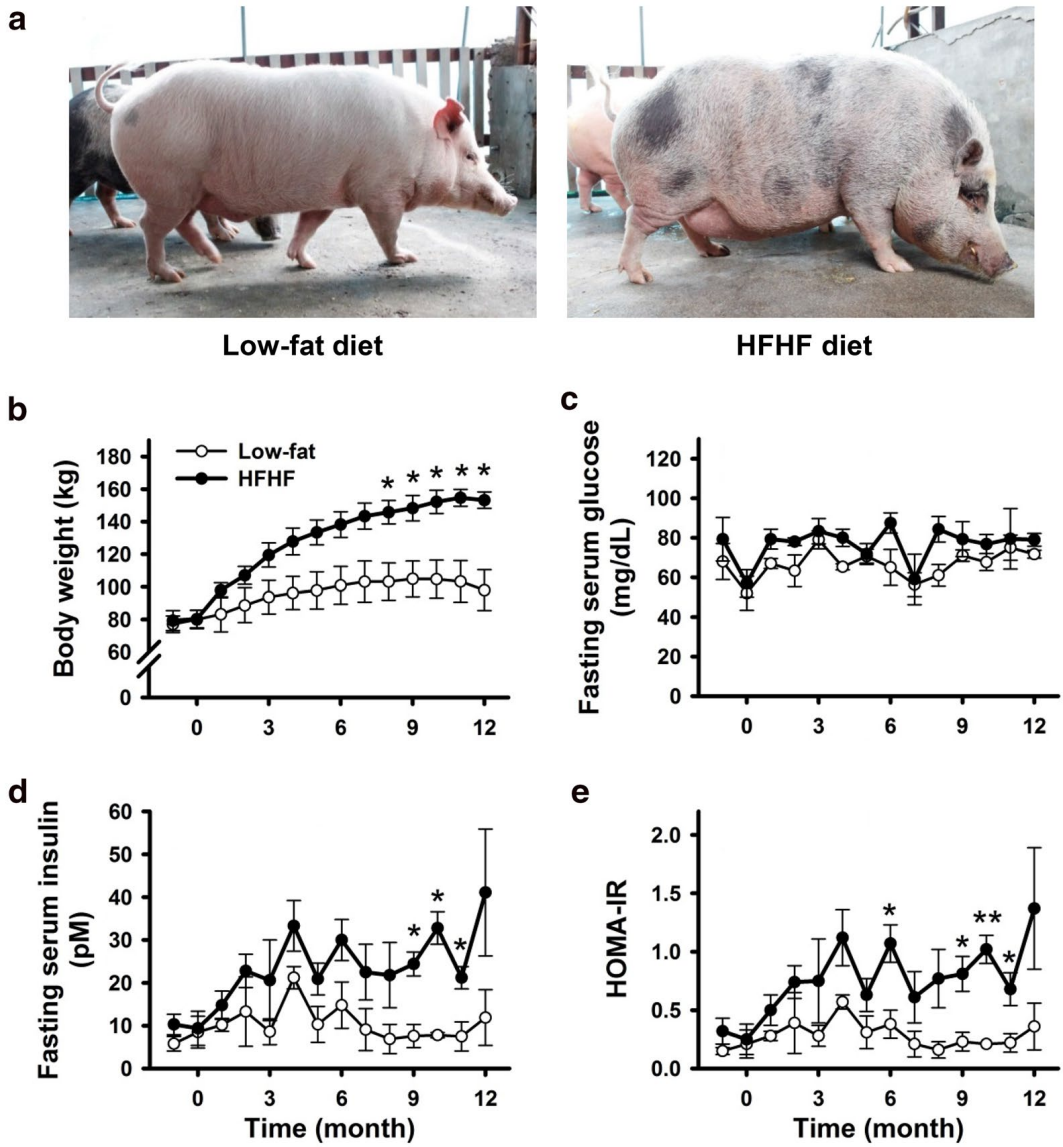
The effects of high-fat plus high-fructose (HFHF) diet on gross appearance, overnight fasting serum glucose and insulin levels, and HOMA-IR in Lee-Sung pigs. Adult male pigs were fed with low-fat or HFHF diets for 12 months. **a** At the end of experiment, HFHF-diet-fed pigs were obese with larger waist sizes, and thicker abdominal fat, as well as backfat and facial fat compared to low-fat diet pigs. During the experimental period, body weight (**b**), overnight fasting serum glucose levels (**c**), fasting serum insulin levels (**d**) and HOMA-IR (**e**) were measured monthly. Data are expressed as means ± SEM (low-fat diet, n = 3; HFHF diet, n = 4). *p < 0.05, **p < 0.01

Lee-Sung pigs fed the HFHF diet gained weight more rapidly than ones fed with low-fat diet (*p* < 0.05) and became obese during the 12-month feeding period (Fig. 1a, b). The body weights of the HFHF group increased mainly during the first 9 months of the experiment then reached a stable plateau during the last 3 months. At the 12th month, the average weight of the HFHF group was 55% heavier than the low-fat diet group (153.2 ± 5.1 vs. 98.0 ± 12.5 kg, respectively, mean ± SEM, *p* < 0.01). In addition, the backfat thickness, determined at the tenth rib, was greater in the HFHF group than in the low fat diet group (4.98 ± 0.13 vs. 3.50 ± 0.45 cm, mean ± SEM, *p* < 0.05). Although the HFHF pigs were obese, the overnight fasting serum glucose was not significantly different compared to the low-fat group (Fig. 1c). In contrast, the fasting serum insulin level in the HFHF pigs was gradually increased during the 12-month feeding period (Fig. 1d).

### Serum biochemical profiles

At the end of the experiment, serum samples were collected and measured for parameters of metabolism and hepatitis (Table 1). The glucose levels of the two groups were not significantly different. However, the HFHF group showed a 7.5 times higher (*p* < 0.05) serum insulin concentration than the low-fat diet group. In serum lipid profiles, the both triglyceride and NEFA levels of the HFHF group were significantly higher than low-fat diet group (*p* < 0.05). Even though total cholesterol levels were similar between groups (*p* > 0.05), there was a tendency toward higher LDL&VLDL-c level in the HFHF group than in the low-fat diet group. Meanwhile, the HDL-c levels in the HFHF group were significantly lower than in the low-fat diet group (*p* < 0.05). Consequently, the HDL-c/LDL&VLDL-c ratio in the HFHF group was significantly lower compared to the low-fat diet group (*p* < 0.05). Dietary fructose consumption induced serum uric acid level increasing has been proposed, and which may advance the development of non-alcoholic fatty liver disease [17]. However, the serum uric acid concentration of Lee-Sung pig is fairly low and below our measuring system. Even though the pigs had high fructose ingestion, the uric acid level couldn’t be measured. Regarding hepatitis parameters, serum AST and ALT were similar between the groups. These data indicated that at 12 months into the feeding regimen the HFHF pigs developed hyperinsulinemia and dyslipidemia but not hyperglycemia or chronic hepatitis.

**Table 1:** The effects of HFHF diet on serum glucose, insulin, triglyceride, NEFA, cholesterol, uric acid, AST and ALT levels in Lee-Sung pigs after 12-month feeding

	Low-fat diet (n = 3)	HFHF diet (n = 4)	HFHF/low-fat (fold)	*p* value
Metabolic parameters				
Glucose (mg/dl)	64.7 ± 3.0	72.3 ± 8.4	1.12	0.490
Insulin (pM)	7.3 ± 1.3	55.0 ± 13.0*	7.50	0.027
Triglyceride (mg/dl)	17.3 ± 3.8	81.0 ± 14.7*	4.68	0.016
NEFA (μM)	32.8 ± 9.7	155.2 ± 17.0**	4.73	0.002
Total cholesterol (mg/dl)	70.0 ± 5.0	69.5 ± 2.9	0.99	0.930
HDL-c (mg/dl)	41.1 ± 4.8	29.8 ± 1.3*	0.73	0.046
LDL&VLDL-c (mg/dl)	43.2 ± 2.5	53.9 ± 3.1	1.25	0.054
HDL/LDL&VLDL ratio	0.95 ± 0.12	0.56 ± 0.05*	0.59	0.019
Uric acid (mg/dl)	< 1.0	< 1.0	–	–
Hepatitis parameters				
AST (IU/l)	12.0 ± 5.0	17.3 ± 9.0	1.44	0.667
ALT (IU/l)	49.3 ± 4.4	34.0 ± 6.4	0.69	0.128

All pigs were fasted 6 h before taking blood samples

*HFHF* high-fat plus high-fructose diet, *NEFA* non-esterified fatty acids, *HDL-c* high density lipoprotein-cholesterol, *LDL&VLDL-c* low density and very low density lipoprotein-cholesterol, *AST* aspartate transaminase, *ALT* alanine transaminase

* *p* < 0.05, ** *p* < 0.01, Data are expressed as means ± SEM (low-fat diet, n = 3; HFHF diet, n = 4)

### Intravascular glucose tolerance test (IVGTT)

**Fig. 2:**
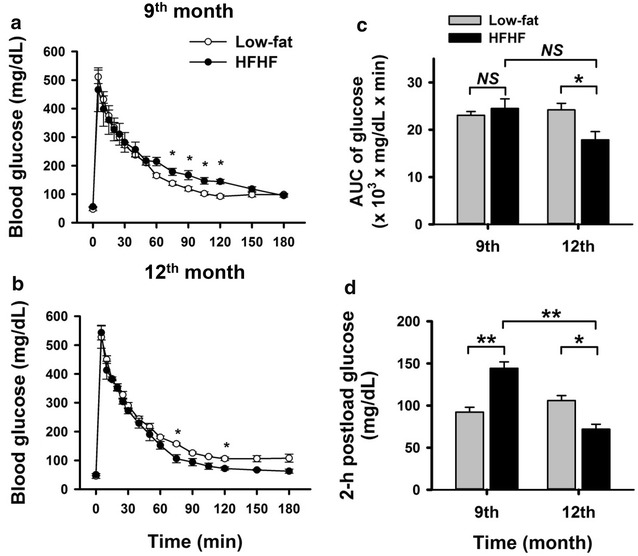
The glucose disposition of intravenous glucose tolerance test (IVGTT) in low-fat and HFHF diet fed pigs. All pigs were subjected to IVGTT at the 9- (**a**) and 12- (**b**) month time points. Briefly, the fasting pigs were injected with glucose solution, 0.5 g/kg body weight, via a jugular vein. For analyzing IVGTT results, the area under the curve (AUC) of glucose level (**c**) and 2-h postload glucose level (**d**) are shown. Data are expressed as means ± SEM (low-fat diet, n = 3; HFHF diet, n = 4). *p < 0.05, **p < 0.01. *NS* not significant

**Fig. 3:**
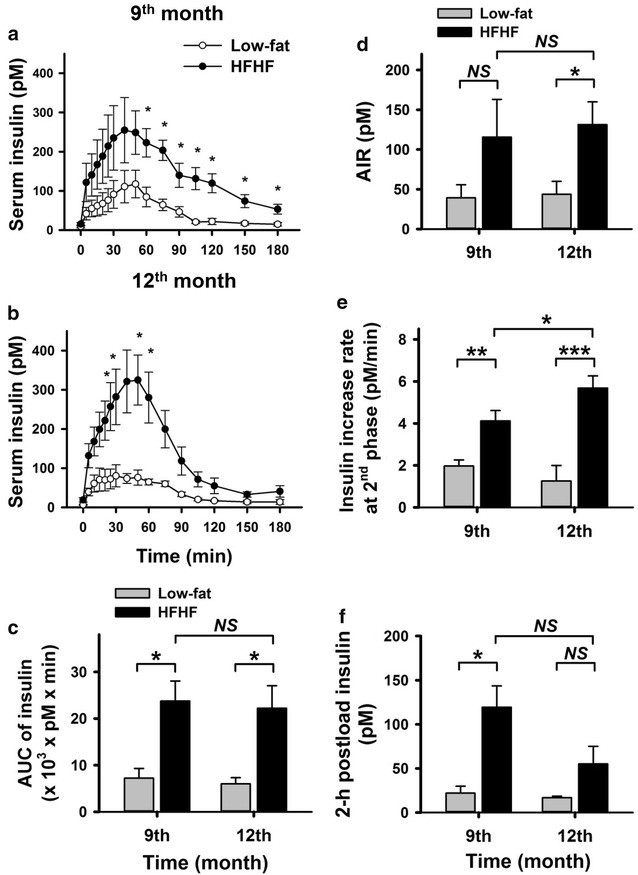
The insulin response to intravenous glucose tolerance test (IVGTT) in low-fat and HFHF diet fed pigs. All pigs were subjected to IVGTT at the 9- (**a**) and 12- (**b**) month time points as in the description in Fig. 2 legend. For analyzing IVGTT results, the area under the curve (AUC) of insulin levels (**c**), acute insulin response (**d**), insulin increase rate at second phase, 10–40 min after glucose injection, (**e**) and 2-h postload insulin level (**f**) are shown. Data are expressed as means ± SEM (low-fat diet, n = 3; HFHF diet, n = 4). *p < 0.05, **p < 0.01, ***p < 0.001. *NS* not significant

The pigs underwent IVGTT for examining glucose tolerance and insulin secretory response during the dietary intervention. The area under of the curve (AUC) of the blood glucose measurements (AUC_glu_) did not differ between the groups at the 9-month test (*p* > 0.05), but the AUC_glu_ of the HFHF group was significantly lower compared to the low-fat group at the 12-month test (*p* < 0.05) (Fig. 2). The insulin level reached a peak at 40–50 min after the glucose injection, and the peak insulin concentrations in the HFHF group were 2–4 times higher than the low-fat diet group (Fig. 3a, b). Furthermore, the AUC of serum insulin was approximately 3–4 times higher in the HFHF group than in the low-fat diet group at the 9- and 12-month tests, respectively (*p* < 0.05) (Fig. 3c). These findings revealed that the HFHF pigs developed mass beta cell hyperactivation and insulin resistance.

Interestingly, the glucose tolerance test showed better result at the 12-month in the HFHF group than at the 9-month, we, thus analyzed other parameters of the IVGTT. The 2-h postload glucose levels in the HFHF group were higher at the 9-month test, with a borderline of IGT (≥ 140 mg/dl) but lower than the low-fat diet group at the 12-month test (*p* < 0.05) (Fig. 2d). At the same time point, the 2-h postload insulin levels in the HFHF group were higher in the 9-month test than in the 12-month test (*p* < 0.05) (Fig. 3f). To determine the cause of these changes, we analyzed the acute insulin response (AIR), which refers to the first phase insulin secretion response after a glucose injection (within 10 min), and the rate of insulin increase at the second phase of insulin secretion (from 10 to 40 min). The results showed that the AIR of the HFHF group was not different between the 9- and 12-month tests (*p* > 0.05) (Fig. 3d), whereas the rate of insulin increase during the second phase was higher (*p* < 0.05) at the 12-month test than the 9-month test (Fig. 3e). Taken together, these data suggested that during the compensation period of prediabetes, the beta cell secretory function of second phase was elevated between the 9- and 12-month time points, and resulted in temporally better glucose disposal.

### Protein kinase B (AKT) phosphorylation in skeletal muscle and liver

For assessment of insulin resistance, homeostatic model assessment (HOMA-IR) was used (Fig. 1e). The HOMA-IR values in the HFHF pigs were gradually increased along with experimental period. Therefore, insulin resistance of the HFHF group was clearly developed.

**Fig. 4:**
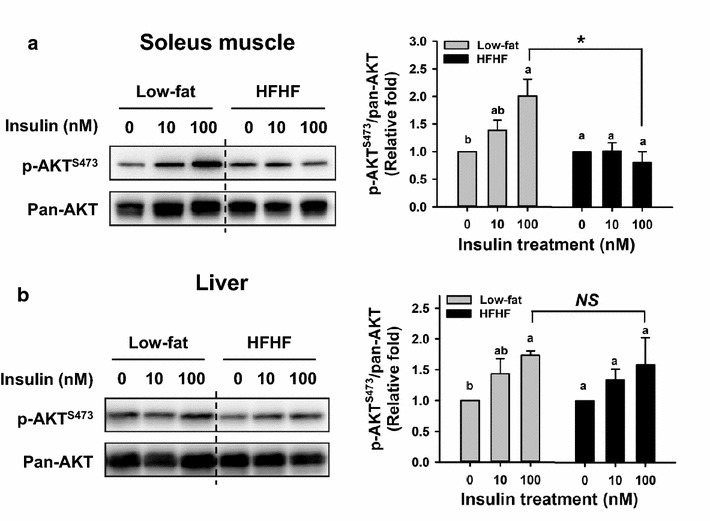
The effect of HFHF diet on the phosphorylation of AKT at Ser473 in skeletal muscle and hepatic tissue after insulin treatment. After the 12-month feeding period, the soleus muscle (**A**) and liver (**B**) specimens were obtained and analyzed for insulin-stimulated phosphorylation levels of protein kinase B (AKT) at S473 ex vivo. The specimens from each pig were treated with different concentrations of porcine insulin (0, 10, and 100 nM) for 30 min and the AKT phosphorylation levels were analyzed by western blot. The bar graph represents the value of p-AKT^S473^/pan-AKT normalized to insulin 0 nM treatment in each pig. Data are expressed as means ± SEM (low-fat diet, n = 3; HFHF diet, n = 4). Data bars with different letters are significantly different in the same dietary group by using LSD test (*p* < 0.05). *p < 0.05 indicates significant difference between two dietary groups on the same concentration treatment by using Student’s t test. *NS* not significant

To further explore insulin resistance in tissues, the AKT Ser473 phosphorylation levels in skeletal muscle and hepatic tissues were examined. AKT, also called protein kinase B, is a downstream intracellular signaling kinase of the insulin receptor. The phosphorylation at Thr308 and Ser473 leads to activation of AKT under insulin stimulation [18]. In ex vivo tests, the muscle tissues from the low-fat diet group showed significantly greater insulin-stimulated phosphorylation responses than the HFHF group (*p* < 0.05) (Fig. 4A). However, in hepatic tissues, insulin-stimulated AKT phosphorylations were similar in both groups (*p* > 0.05) (Fig. 4B). These data indicated that insulin resistance in the HFHF pigs may have occurred earlier in skeletal muscle than liver.

## Discussion

In the current study, we investigated the progressive changes in insulin secretory response and the development of tissue-specific insulin resistance in the HFHF diet-fed adult Taiwan Lee-Sung miniature pigs. According to the results, the pigs presented several features, including normal fasting glycemia, hyperinsulinemia and dyslipidemia, similar to insulin-resistant or IGT prediabetic subjects [8, 19]. In addition, the pigs also displayed intense insulin secretion, glucose intolerance and skeletal muscle insulin resistance. With supporting the hypothesis, the insulin resistance developed in skeletal muscle prior to liver tissues in the HFHF pigs.

A prospective study of over 6500 subjects with 9.7 years follow-up indicated that those who developed diabetes had higher fasting and 2-h postload glucose, fasting and 2-h postload insulin, and beta cell secretory function, and lower HOMA-IR insulin sensitivity compared to non-diabetes subjects at baseline before diagnosis [20]. Most of these features were confirmed in the current study of pigs subjected to long-term feeding of the HFHF diet. The HFHF pigs showed normal fasting glucose and higher 2-h postload glucose and muscle insulin resistance (lack of AKT Ser473 phosphorylation), which suggested that these pigs had isolated IGT. In addition, the high level of fasting insulin concentration was clearly observed in insulin-resistant obese or prediabetic individuals in the numerous clinical reports and current study [3, 8, 19–21]. Dyslipidemia, with higher serum triglyceride, and lower serum HDL-c levels, is a strong correlative to insulin resistance [8, 21]. Several studies suggested that lipids could induce insulin resistance in liver and skeletal muscle by interfering with downstream signal transduction of the insulin receptor [22–25]. To compensate for tissue insulin resistance, the basal insulin level should elevate to maintain blood glucose homeostasis. Lipids also may contribute to basal insulin hypersecretion, due to an increase of intracellular reactive oxygen species in the beta cell [26, 27]. In addition to lipids, fructose is purposed to enhance glucose-stimulated insulin secretion via the sweet taste receptor on the beta cell, thereby amplifying the intracellular calcium signal [28]. Taken together, hyperinsulinemia, obviously present in the HFHF pigs, was caused by multiple factors including indirect increasing peripheral insulin resistance and direct stimulating beta cell function.

The pancreatic insulin secretory capacity changes during the long period of prediabetes. Tabák et al. indicated that the insulin secretion in diabetic subjects began increasing between 4 and 3 years before diagnosis, with a steep decrease beginning 3 years before diagnosis, the late stage of prediabetes [20]. Therefore, unlike the descriptions from previous studies that prediabetic subjects have decreasing insulin secretory responses [5, 6], the HFHF pigs showed excessive secretion of insulin by IVGTT during the early stage of prediabetes. In addition, the temporary amelioration of glucose tolerance by way of increasing the 2nd phase insulin secretion was observed at the 12-month time point. These results supported the idea that elevating insulin secretory capacity to compensate the developing insulin resistance on the prediabetic status. Of note, the assessment of insulin secretion in these studies was based on the insulinogenic index, the ratio of change in plasma insulin and glucose levels during glucose challenge (ΔIns/ΔPG) [5, 6]. This index is more suited to pancreatic tissue and cell culture compared to whole-body in vivo measurements because the glucose disposal rate effects change plasma glucose levels and should be considered. In addition, the observation of increased 2-h postload insulin in the subjects with isolated IGT or combined IFG/IGT in these studies also supported our finding.

Ectopic lipid accumulation has been considered as a critical pathogenic factor for developing insulin resistance in both muscular and hepatic tissue [29]. The cause of ectopic lipid accumulation may be the loss of lipid-buffering capacity of insulin resistance-adipose tissues, which leads to lipolysis and elevated circulating lipid levels. The current study supports this hypothesis, which the HFHF pigs had the high level of serum NEFA and dyslipidemia. The ectopic lipid accumulation in the both skeletal muscle and hepatic tissue were also observed (Additional file 1: Figure S1). However, the tissue triglyceride content was not absolutely related to the development of insulin resistance, because only the skeletal muscle had progressed to decreasing insulin sensitivity. Furthermore, instead of triglyceride, the diacylglycerol and ceramide are regarded as the key factors in aggravating insulin resistance [29]. These lipid metabolites disrupt insulin signaling transduction by activating the several isoforms of protein kinase Cs (PKCs), including PKCθ PKCε and PKCζ [29]. On the basis of the ex vivo testing of insulin-stimulated AKT Ser473 phosphorylation, insulin resistance was primarily observed in skeletal muscle in the HFHF pigs. This evidence also supports the idea that insulin resistance in skeletal muscle rather than liver is the preliminary defect in the development of metabolism syndrome and type 2 diabetes [9, 30]. Therefore, we proposed that adipose tissue initially has insulin resistance and increasing lipolysis, which leads to ectopic lipid accumulation in both skeletal muscle and liver. The skeletal muscle is more rapid in developing insulin resistance than liver, and precipitates the deterioration in glucose tolerance.

Fructose consumption is a potential factor that induced hepatic *de novo* lipogenesis resulting in non-alcoholic fatty liver disease and hepatic insulin resistance [17, 31, 32]. Unexpectedly, the HFHF pigs did not develop fatty liver (as assessed by histological analysis), tremendous hepatic triglyceride content (Additional file 1: Figures S1, S2), hyperuricemia, and hyperglycemia, the outcomes of elevated endogenous glucose production under conditions of hepatic insulin resistance. Clinical studies have indicated that only small amounts, 0.05 and 0.15%, of absorbed fructose are converted de novo to fatty acids and triglyceride-glycerol, respectively [33], and most, 28.9–37.4%, is converted to glucose [34]. However, fructose consumption indeed increases newly synthesized triglyceride-glycerol in circulating VLDL [33], which suggests that fructose may alter hepatic lipid metabolism through other mechanisms instead of direct contribution to lipid synthesis. It has been demonstrated that uric acid mediates fructose-induced lipid accumulation in hepatocytes [35]. Metabolizing fructose transiently depletes intracellular ATP and results in uric acid overproduction, which causes mitochondrial oxidative stress and de novo lipogenesis. Unlike the human, deficient in functional uricase, most mammalians are able to metabolize uric acid and therefore have relatively low level of uric acid in circulation [36]. In the context of the present study, the metabolism of fructose and uric acid in the pigs may be different than humans, resulting in a lack of fructose-induced hyperuricemia, hepatic steatosis and insulin resistance.

Prediabetes contains several features with insulin resistance, hyperinsulinemia, glucose intolerance and/or hyperglycemia. According to the previous studies and current data, swine is the species that could be induced to hyperinsulinemia but hardly to hyperglycemia under the excess fat and energy dietary intervention (Table 2). Several miniature pig strains, including Göttingen [37, 38], Ossabaw [14, 39] and Yucatan [40], had various degrees of increasing fasting insulin levels or insulin hypersecretion in the glucose tolerance test after high-fat diet feeding for 3–6 months. However, the fasting glucose levels were shown as slight or no increase. A recent study, using Bama miniature pigs fed with high-fat and high-sucrose diet for 23 months, displays the gradually increasing fasting insulin levels and glucose intolerance along with the lateral-half experimental period [41]. In spite of long-term feeding, the hyperglycemia was not developed in these pigs. Therefore, swine fed with high-fat and high-energy diet could present IGT-like prediabetes. Additionally, on the basis of above studies and present results, we suggest that the longer dietary intervenient period rather than porcine strain is the major factor to result in more severe hyperinsulinemia and insulin resistance. It has been reported that hyperglycemic porcine model by using chemical induction, such as streptozotocin and alloxan, is established [10, 11]. Unlike dietary intervention model, these pigs lack hyperinsulinemia and insulin resistance. Due to hyperinsulinemia and insulin resistance relating to various disease developments [40, 41], the dietary intervention model may be more applicable to study IGT-type prediabetic pathophysiology and prophylaxis.

**Table 2:** The comparisons of metabolic manifestations in different porcine strains and dietary interventions

Strain	Age	Intervention		Body weight (kg)	Fasting glucose level	Fasting insulin level	Features	Refs.
		Diet	Time					
Lee-Sung	2 years	High fat high fructose	12 months	Con: 98 Int: 153 (1.6 ×)^a^	Con: 3.9 mM Int: 4.4 mM (1.1 ×)	Con: 2.0 μIU/ml Int: 6.9 μIU/ml (3.5 ×)	Hyperinsulinemia, dyslipidemia, insulin resistance, intense insulin secretion (IVGTT)	Current
Ossabaw	5–10 months	High fat high fructose^b^	6 months	Con: 56 Int: 87 (1.6 ×)	Con: 77 mg/dl Int: 87 mg/dl (1.1 ×)	Con: 10 Int: 18 (1.8 ×)	Slight hyperinsulinemia, severe dyslipidemia, insulin resistance	[14]
Ossabaw	6 months	High fat high fructose^b^	6 months	Con: 54 Int: 91 (1.7 ×)	Con: 73 mg/dl Int: 80 mg/dl (1.1 ×)	Con: 5 μIU/ml Int: 21 μIU/ml (4.2 ×)	Hyperinsulinemia, severe dyslipidemia, insulin resistance	[39]
Göttingen	7–8 months	High fat high energy	3 months	Con: 24 Int: 32 (1.3 ×)	Con: 3.6 mM Int: 4.3 mM (1.2 ×)	Con: 23 pM Int: 80 pM (3.5 ×)	Hyperinsulinemia, slightly increasing insulin secretion (IVGTT)	[37]
Göttingen	7 weeks	High fat high sucrose	4 months	Con: ~ 14 Int: ~ 17 (1.2 ×)	Con: 4.2 mM Int: 4.7 mM (1.1 ×)	Con: 34 pM Int: 47 pM (1.4 ×)	Slight hyperinsulinemia, dyslipidemia, slightly increasing insulin secretion (IVGTT)	[38]
Yucatan	3 months	High fat high fructose	7 months	Con: 40 Int: 73 (1.8 ×)	Con: ~ 2.3 mM^b^ Int: ~ 2.8 mM^c^ (1.2 ×)	Con: ~ 1.5 μIU/ml^c^ Int: ~ 5 μIU/ml^c^ (3.3 ×)	Hyperinsulinemia, dyslipidemia, insulin resistance, intense insulin secretion (IVGTT)	[40]
Bama	6 months	High fat high sucrose	23 months	Con: 51 Int: 140 (2.7 ×)	Con: 6.62 mM Int: 4.99 mM (0.8 ×)	Con: 5.7 μIU/ml Int: 28.3 μIU/ml (5 ×)	Hyperinsulinemia, dyslipidemia, insulin resistance	[41]

*Con* control diet group, *Int* intervention diet group

^a^The number in parentheses is the fold value between two groups

^b^Supplied with additional cholesterol and sodium cholate

^c^The value is estimated from dot graph

## Conclusion

In summary, Taiwan Lee-Sung miniature pigs fed a HFHF diet for 12 months developed several features similar to insulin-resistant or IGT prediabetic subjects. These features included normal fasting glucose, dyslipidemia, glucose intolerance, insulin hypersecretion after glucose challenge, and skeletal muscle insulin resistance. While in the initial stage of prediabetes, the insulin secretory capacity was increased to compensate for the developing insulin resistance. On the basis of the similarity with clinical pathological features, this porcine model could provide a useful platform to explore the pathophysiology of prediabetes and to examine novel therapeutic interventions to prevent type 2 diabetes.

## Additional file

**Additional file 1: Table S1.** Compositions of experimental diets. **Figure S1.** The effect of HFHF diet on the triglyceride accumulations in the skeletal muscle and hepatic tissues. Figure S2. Liver histology of Lee-Sung pigs at the end of the dietary intervention.

## Abbreviations

AIR: acute insulin response
AKT: protein kinase B
AUC: area under of the curve
HFHF: high-fat plus high-fructose diet
HOMA-IR: homeostatic model assessment
IFG: impaired fasting glucose
IGT: impaired glucose tolerance
IVGTT: intravenous glucose tolerance test
NGT: normal glucose tolerance
