# Supplementary material for: Long-term feeding of high-fat plus high-fructose diet induces isolated impaired glucose tolerance and skeletal muscle insulin resistance in miniature pigs
Source: Diabetol Metab Syndr. 2017 Oct 13;9:81. doi: 10.1186/s13098-017-0281-6 (PMC5640912; doi:10.1186/s13098-017-0281-6)
Supplement: Supplementary file 1 — Additional file 1: Table S1. Compositions of experimental diets. Figure S1. The effect of HFHF diet on the triglyceride accumulations in the skeletal muscle and hepatic tissues. Figure S2. Liver histology of Lee-Sung pigs at the end of the dietary intervention. [file 13098_2017_281_MOESM1_ESM.pdf]

Additional Information

Tissue triglyceride measurement

Small pieces (about 100 mg) of tissue were homogenized in 2 ml chloroform:methanol (2:1) using tissue homogenizer for at least 10 min and incubated 1 hr at room temperature on the orbital shaker. The homogenates were centrifuged at 5000 xg for 5 min, and the liquid phase was transferred to new tube. The extract was mixed well with 0.4 ml saline and centrifuged 200 xg for 10 min. the lower organic phase was transferred to the new tube and evaporated organic solvent at 50 ℃ by using the dry bath. Finally, the lipid extract was resolved in methanol containing 10% triton X-100 and analyzed triglyceride concentration by using triglyceride determination kit (Sigma).

Histological analysis

For hematoxylin and eosin (H&E) staining, liver tissue specimen was fixed in neutral-buffered formalin solution (10% formalin in 1X phosphate-buffer saline), embedded in paraffin, and processed to 4-μm thickness sections. The re-hydrated slides were stained with hematoxylin solution Gill III (Merck Millipore, Billerica, MA, USA) and 0.5% Eosin Y-solution (Merck Millipore) as manual description. Finally, slides were rinsed in tap water and hydrated with ethanol and xylene prior to adding mounting medium (Hecht-Assistent; Sondheim, Germany). For glycogen staining, periodic acid-Schiff (PAS) stain was used. The liver specimen sections were prepared as above description and followed the procedures of PAS staining kit (Sigma) manual. The slides were further counterstained with hematoxylin for 30 sec, and hydrated with ethanol and xylene prior to adding mounting medium. For Oil Red O staining, fresh liver specimen was embeded in Tissue-Tek® O.C.T. Compound (Sakura Fineteck USA, Inc., Torrance, CA, USA) and sectioned to 7-μm thickness by using freezing microtome. The sections were further fixed in 3.7% formaldehyde, rinsed with deionized water, stained with Oil Red O working solution (containing 0.3% Oil Red O (Sigma) and 36% triethyl phosphate(Sigma)) for 30 min, then rinsed with tap water for 5 min. Finally, sections were counterstained with hematoxylin and mounted with 10% glycerol solution. All slides were observed with an Axioskop 40 microscope (Carl Zeiss, Göttingen, Germany) equipped with digital camera AxioCam ERc 5s (Carl Zeiss).

Table S1. Compositions of experimental diets

Ingredient	Low-fat diet (3265 Kcal/Kg)[#] (%)	High-fat plus high-fructose diet (3950 Kcal/Kg)[#] (%)
Corn meal	77.14	19.33
Soybean (seed, heat processed)	11.38	43.32
Fish meal (herring)	5.00	0.00
Cellulose	3.00	3.50
Fructose	0.00	17.80
Lard	0.98	12.66
$Ca(H_2PO4)_2$	0.62	0.76
$CaCO_3$	1.08	1.76
NaCl	0.30	0.36
Vitamins	0.30	0.30
Minerals	0.20	0.20
**Energy Source**		
From protein	15.8	15.7
From fat	17.8	45.0
From Carbohydrates	66.4	39.2

[#] Dietary metabolizable energy

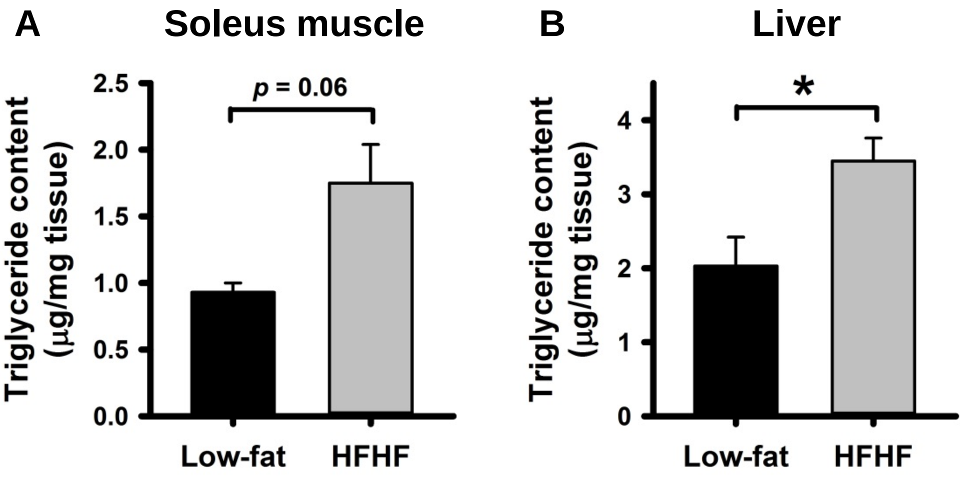

Figure S1. The effect of HFHF diet on the triglyceride accumulations in the skeletal muscle and hepatic tissues. After the 12-month experimental period, the porcine soleus muscle (A) and liver (B) specimens were subjected to lipid extraction and analyzed for tissue triglyceride content. The results show that the HFHF pigs had higher triglyceride accumulation in both muscle and liver tissues. However, the HFHF pigs could not be diagnosed as having fatty liver, which defined as the lipid content greater than 5% of liver weight. Data are expressed as means ± SEM (Low-fat diet, n = 3; HFHF diet, n = 4). *$p < 0.05$.

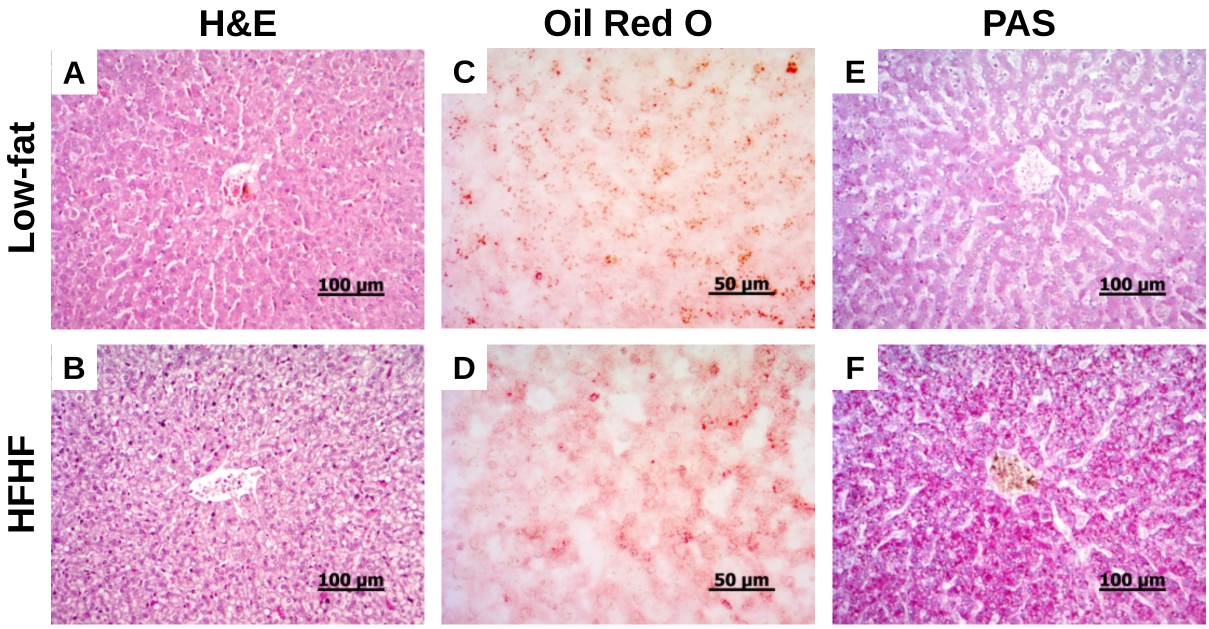

Figure S2. Liver histology of Lee-Sung pigs at the end of the dietary intervention. In the hematoxylin and eosin (H&E) staining results (A and B), the HFHF group showed irregular mosaic white pattern rather than foamy pattern in the cells, which suggests extensive glycogen storage. To further confirm the observation, oil red O staining (C and D) and periodic acid-Schiff (PAS) staining (E and F) were examined in the frozen- and paraffin-section, respectively. The glycogen deposit (magenta color) manifestly in the HFHF group, but the lipid droplet distribution (red color) are not different between two groups. According to these histological analysis, the pigs did not develop severe non-alcoholic fatty liver disease after long-term HFHF diet feeding.
